# Recent advances in understanding and management of bronchopulmonary dysplasia

**DOI:** 10.12688/f1000research.25338.1

**Published:** 2020-07-14

**Authors:** Mitali Sahni, Vineet Bhandari

**Affiliations:** 1 Pediatrix Medical Group, Sunrise Children’s Hospital, Las Vegas, NV, USA; 2 University of Nevada, Las Vegas, NV, USA; 3 Neonatology Research Laboratory, Education and Research Building, Cooper University Hospital, Camden, NJ, USA

**Keywords:** Newborn, hyperoxia, chronic lung disease, premature

## Abstract

In the current era, the survival of extremely low-birth-weight infants has increased considerably because of new advances in technology; however, these infants often develop chronic dysfunction of the lung, which is called bronchopulmonary dysplasia (BPD). BPD remains an important cause of neonatal mortality and morbidity despite newer and gentler modes of ventilation. BPD results from the exposure of immature lungs to various antenatal and postnatal factors that lead to an impairment in lung development and aberrant growth of lung parenchyma and vasculature. However, we still struggle with a uniform definition for BPD that can help predict various short- and long-term pulmonary outcomes. With new research, our understanding of the pathobiology of this disease has evolved, and many new mechanisms of lung injury and repair are now known. By utilizing the novel ‘omic’ approaches in BPD, we have now identified various factors in the disease process that may act as novel therapeutic targets in the future. New investigational agents being explored for the management and prevention of BPD include mesenchymal stem cell therapy and insulin-like growth factor 1. Despite this, many questions remain unanswered and require further research to improve the outcomes of premature infants with BPD.

## Introduction

Bronchopulmonary dysplasia (BPD) was described as a new lung disease in preterm infants with respiratory distress syndrome (RDS) by Northway *et al*., in 1967 ^1^. They described this syndrome in premature infants exposed to high supplemental oxygen and invasive mechanical ventilation who developed lung injury with specific airway histopathological features. BPD has evolved over the past five decades with the advent of new and effective perinatal strategies, including the use of antenatal steroids and surfactants, restricted use of oxygen, and a focus on the use of non-invasive, gentler modes of ventilation ^2^. Despite this, BPD remains one of the most common complications associated with prematurity ^3^. Newer BPD tends to have a milder initial clinical presentation of the illness compared to old BPD. A subgroup of infants with new BPD are ventilator-dependent and develop severe disease as it moves from the early to evolving and established phases ^4, 5^. BPD continues to be a major cause of respiratory morbidities in the modern era as we push the limits of viability and see increased survival of extremely preterm infants born at 22–24 weeks of gestation ^6^. In this review, we discuss the new advances in understanding the pathobiology of this disease, the current controversy regarding the definition of BPD, use of the novel “omic” approaches in BPD, and how this affects our assessment of this disease. We also discuss the current and upcoming therapies for the management of BPD and summarize the unanswered questions that require further research to improve the outcomes of premature infants with BPD.

## Epidemiology and pathophysiology

Based on studies from various neonatal networks across many countries, including the United States, Canada, Korea, China, and India, the prevalence of BPD has been reported to be quite variable, ranging from 11 to 50%, and can be attributed to differences in the criteria of diagnosis and management approaches ^7^. The incidence of BPD increases with a decrease in gestational age (GA) and birth weight ^6^. The other perinatal factors affecting it are male sex ^8^, intrauterine growth restriction ^9^, chorioamnionitis ^10^, smoking ^11^, and race/ethnicity ^9^. Postnatal factors also increase the risk for BPD and poor outcomes, including RDS at birth, requirement for invasive mechanical ventilation and supplemental oxygen, lung inflammation, pulmonary vascular disease, air leaks, infections, presence of hemodynamically significant patent ductus arteriosus, and nutritional deficiencies ^12^.

A potential role for placental insufficiency in the development of BPD and BPD-associated pulmonary hypertension (PH) has been proposed and investigated in recent years. Placenta-mediated complications in pregnancy are associated with moderate to severe BPD in very preterm infants. This was independent of GA and birth weight ^13^. Fetal growth restriction can predispose to impaired lung development. Similarly, decreased placental villous vascularity was seen in BPD-associated PH ^14, 15^. These studies show that placental disturbances play a vital role in lung alveolarization and represent a novel and important area in lung developmental pathobiology ^16, 17^.

**Figure 1.**
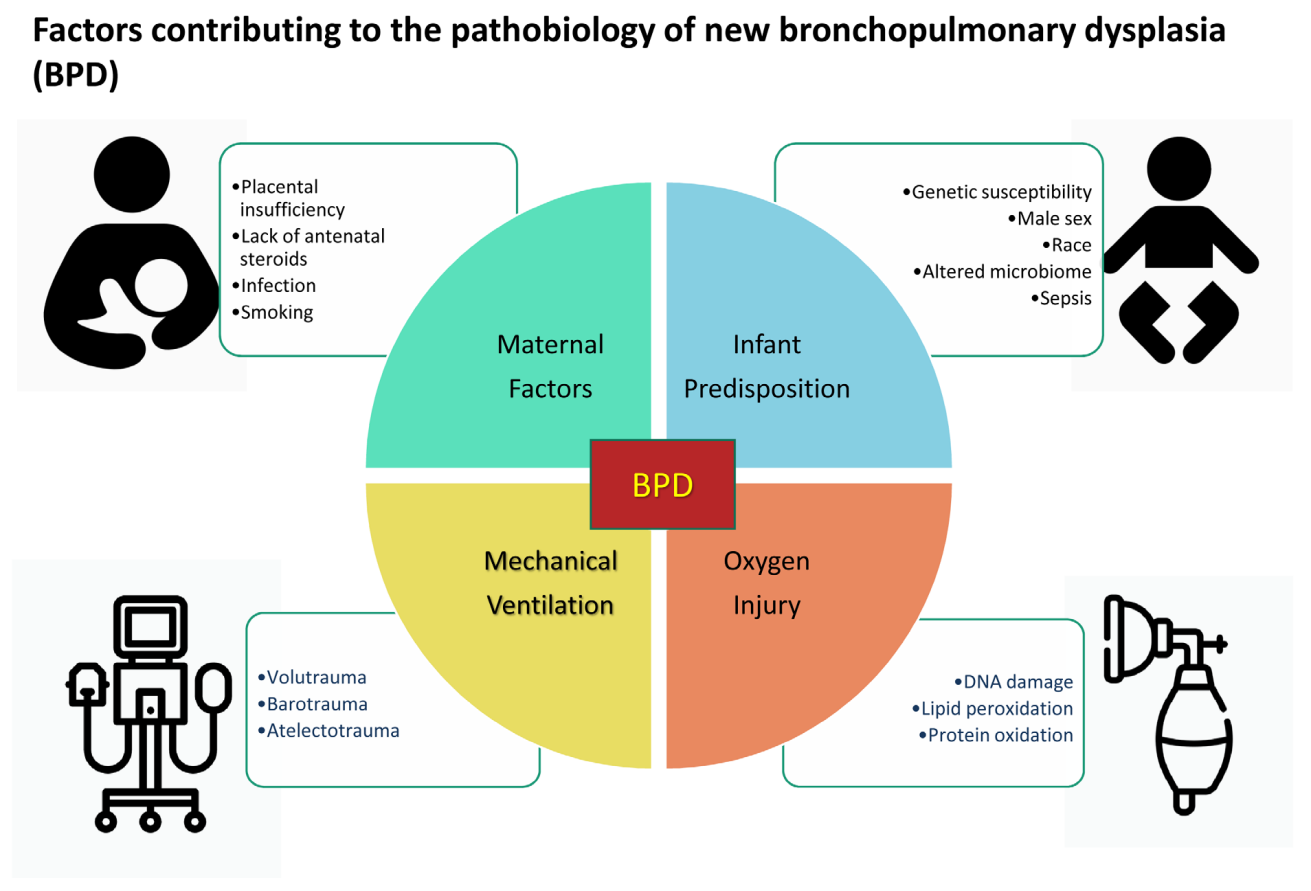
Factors contributing to the pathobiology of new bronchopulmonary dysplasia (BPD). Premature infants at risk owing to maternal and infant factors develop new BPD when exposed to invasive mechanical ventilation and hyperoxia.

The microbiome is also emerging as an important factor in disease pathobiology, and studies have revealed differences in the airway microbiome of preterm and term infants and infants with BPD. Furthermore, longitudinal changes in the airway microbiome of preterm infants requiring invasive mechanical ventilation may be associated with severity of BPD, although it is yet to be determined if these changes are causal or a consequence of the management of these infants. Preterm infants <34 weeks GA and birth weight 500–1,250 g who eventually developed severe BPD demonstrated abundance of *Ureaplasma* and less *Staphylococcus* in their tracheal aspirates in the first few days after birth ^18, 19^. All of these studies attest to the potential contribution of the microbiome in the dysregulated development of the lungs ^20, 21^. Figure 1 summarizes the factors contributing to the pathobiology of new BPD.

## Diagnosis and assessment

### Does an ideal definition of bronchopulmonary dysplasia exist?

Various definitions exist for diagnosing BPD; however, most of them suffer from the primary limitation of utilizing a treatment modality as the primary factor defining the disease rather than the disease pathology or severity ^22^. One of the commonly used definitions of BPD is the NIH consensus definition by Jobe *et al*., which recommends diagnosing infants with BPD based on their oxygen exposure and determines severity by assessing their respiratory support at 36 weeks postmenstrual age (PMA) ^23^. A major limitation of the available definitions of BPD is their inability to diagnose BPD in infants dying from severe respiratory failure before 36 weeks PMA. These infants comprise a subset of extremely preterm infants who are at high risk for developing severe BPD if they survive. An additional challenge comes from the increasing use of high-flow nasal cannula for oxygen therapy that provides a variable degree of positive end-expiratory pressure, thus creating the conundrum of whether these infants should be classified on the basis of supplemental oxygen concentration alone or by including the positive pressure that they receive. On the other end of the spectrum, we have infants on 100% oxygen via very low nasal cannula flows who may not be appropriately categorized with the available definitions. Another group of infants difficult to classify are those dependent on positive-pressure ventilation for airway abnormalities or abnormal respiratory control with no or minimal parenchymal lung disease. These infants should not be classified as having BPD, but we do not have the adequate means to re-categorize them currently ^24^.

In the summary from the workshop on BPD organized by the *Eunice Kennedy Shriver* National Institute of Child Health and Human Development (NICHD), the expert panel proposed an updated definition of BPD ^25^. In this new definition, the panel addresses the use of contemporary modes of ventilation and stage BPD based on the precise respiratory support and fraction of inspired oxygen (FiO _2_) being used. They also exclude the need for supplemental O _2_ exposure for 28 days and include “radiographic confirmation of parenchymal disease” ^26^. The use of this definition accurately predicted death or serious respiratory morbidity at 18–26 months corrected age in 81% of infants in the study ^27^. An ideal definition of BPD should be objective, take into account the pathophysiology, and be able to predict meaningful long-term outcomes ^28, 29^. Use of long-term biomarkers for assessing lung injury ^30^, imaging studies like functional magnetic resonance imaging to assess lung structure ^31^, and novel ways to assess pulmonary function will be required to create a new classification system for BPD that is more centered on lung function evaluation and pathophysiology ^28^.

### The “omics” in bronchopulmonary dysplasia

In order to accurately assess the lung pathology in BPD, several traditional biomarkers have been utilized, but, owing to variable phenotypes seen in BPD, these biomarkers have low predictive value ^30^. In the past decade, newer technologies have allowed the detailed analysis of “big data sets” through bioinformatics to help in delineating the pathogenesis, mechanisms, and diagnosis of diseases. Novel systems biology-based “omic” approaches can be utilized to define the multiple cellular and humoral interactions that regulate the dysregulated lung development and injury response in BPD.

Metabolomics has been utilized to develop a panel of biomarkers to identify neonates at risk of developing BPD. Metabolomics consists of quantitative analysis of many low-molecular-mass metabolites found in a specific cell, organ, or organism involving substrates or products of a defined metabolic pathway. The changes in metabolite composition reflect the interaction among a genetic predisposition, a specific pathophysiological state, and environmental stimuli and can provide a unique fingerprint of those pathophysiological changes that predict the predisposition to the disease ^32^. La Frano *et al*., studied the metabolomics in umbilical cord blood from 10 neonates who later developed BPD and compared them to 10 controls and found an alteration in lipid metabolism that may be responsible for an immaturity of lipid biosynthesis in these infants ^33^. In another study evaluating tracheal aspirate fluid from 68 preterm neonates on the first day of life, the authors demonstrated that neonates who develop BPD later in life have higher levels of serine, alanine, taurine, and citrulline compared to neonates who do not develop BPD ^34^.

As mentioned earlier in this review, various studies have demonstrated associations between dysbiosis of the airway microbiome and BPD; however, the direction of causality between airway injury during lung development and respiratory colonization with microorganisms is unclear. A systematic review of six studies investigating the airway microbiome of preterm infants showed that early microbiome contains Firmicutes and Proteobacteria as the predominant phyla and *Staphylococcus* and *Ureaplasma* as the predominant genera ^19^. Infants who eventually developed severe BPD demonstrated an abundance of *Ureaplasma* and less *Staphylococcus* in their tracheal aspirates initially after birth ^18^. BPD progression was associated with increased microbial community turnover, change in relative abundance of *Firmicutes* and *Proteobacteria*, and decrease in *Lactobacilli* ^19, 20^. Metabolomics and microbiomics can be integrated to help guide us to the pathways that are activated in newborns who develop BPD based on the microbiome they have in their airways/lungs ^32^. Lal *et al*. investigated this relationship between airway microbiome and metabolome at birth. They found that in infants with BPD, there is an increase in *Proteobacteria*, a reduction in *Lactobacilli*, and a decrease in the ratio of acetyl-CoA/propionyl-CoA carboxylase, indicating a curtailed fatty acid β-oxidation pathway. These changes suggest that the metabolic activity of the airway microbiome may in fact modulate the metabolome. The authors further postulated that the lipid metabolites from the bacteria may cause increased airway inflammation, leading to BPD ^35^.

Proteomics is the large-scale study of the structure and function of proteins which may help evaluate the networks of proteins that can specify the real-time status of disease state and protein function modulation ^36^. Analysis of bronchoalveolar lavage fluid in preterm babies using a proteomics-based approach showed that calcium signaling-related proteins differed with BPD severity ^37^. In a study evaluating plasma proteins using proteomics in preterm infants, it was noted that there was decreased gelsolin, afamin, and carboxypeptidase N subunit 2 levels in cord blood ^38^. In addition, there was decreased hemoglobin subunit gamma-1 and galectin-3 binding protein levels, along with increased serotransferrin in plasma at 36 weeks PMA ^38^. In a recent study by Förster *et al*., proteomic screening was utilized in plasma samples from preterm infants to identify novel biomarkers of BPD that could help in the early identification of such infants. They reported that increased levels of sialic acid-binding immunoglobulin-type lectin (SIGLEC)-14 and basal cell adhesion molecule (BCAM) along with decreased levels of angiopoietin-like 3 (ANGPTL3) in the first week of life can predict the outcome of BPD with high specificity and sensitivity ^39^.

In the field of genomics, various approaches including integrated genomic analysis ^40^ and gene expression profiling ^41^ have led to the identification of novel pathways of lung development and repair (the cell-surface glycoprotein CD44, phosphorus oxygen lyase activity) and novel molecules and pathways (adenosine deaminase, targets of microRNA [miR]) that are associated with the genetic origins of BPD ^42^. In addition to whole exome sequencing and whole genome sequencing to identify non-common variants in patients with BPD, RNA sequencing methods have been developed to evaluate protein-coding mRNA, transcripts of pseudogenes, long non-coding RNA (lncRNA), and miRs ^43^. In the last few years, miRs have been described as important mediators of normal growth, development, and disease. In a recent study, Syed *et al*. showed that miR-34a levels are significantly increased in the lungs of neonatal mice exposed to hyperoxia; deletion or inhibition of miR-34a improved the pulmonary phenotype and BPD-associated pulmonary arterial hypertension (PAH) in mouse models of BPD ^44^. Pharmacologic inhibition of miR-34a can thus be used as a potential therapeutic option in neonates to prevent hyperoxia-induced lung injury ^45^. In another study, increased expression of miR-451 was noted in animal models of BPD and inhibition of miR-451 was shown to improve the cardiopulmonary phenotype ^46^. Similarly, potential roles for miR-17∼92 ^47^, miR-29b ^48^, miR-876-3p ^49^, miR-199a-5p ^50^, and miR-489 ^51^ have been described in the pathogenesis of BPD.

## New and upcoming therapies in the management and prevention of bronchopulmonary dysplasia

**Figure 2.**
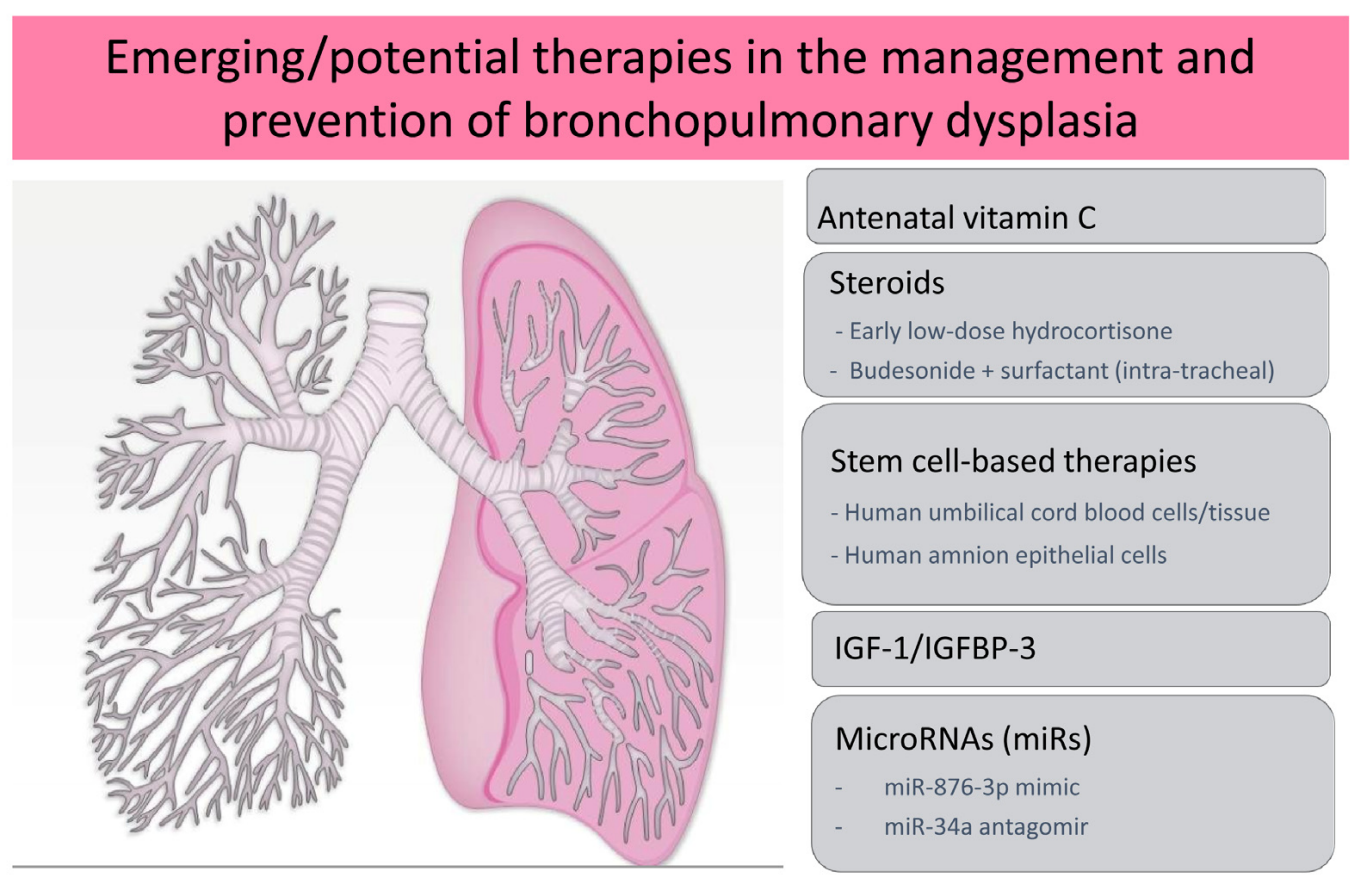
Emerging/potential therapies in the management and prevention of bronchopulmonary dysplasia. These include antenatal vitamin C, steroids, stem cell-based therapies, insulin-like growth factor-1 (IGF-1) in combination with IGF-binding protein-3 (IGFBP-3), and microRNAs.

Despite the high prevalence of BPD, no new drugs have been approved for the management of this disease over the past two decades. To date, among the many tried interventions, postnatal use of vitamin A ^52, 53^, caffeine ^54^, and corticosteroids ^55^ are the only interventions that have led to a decrease in the rate of BPD. The use of vitamin C in pregnant smokers has been shown to improve pulmonary function tests in newborns at birth and 3 months of age with decreased wheezing up to 1 year of age ^56, 57^. Since smoking is a significant risk factor for BPD, use of vitamin C in this population may help to mitigate the effects of smoking on the developing lung; however, no clinical trials have evaluated this potential therapy as yet specifically to decrease BPD. Systemic corticosteroids have been used for decades to decrease BPD, with dexamethasone being one of the initial therapies to have been adopted. Although dexamethasone clearly decreased the rate of BPD, it caused various short- and long-term adverse effects that have led to a decrease in its use over time ^58^. Dexamethasone lacks mineralocorticoid activity and has been associated with serious neurodevelopmental impairment due to apoptosis in areas of the brain critical to learning and memory ^59^. Over the past few years, hydrocortisone use has gained popularity in extremely premature infants either for the management of hemodynamic failure or for the prevention of BPD ^55^. An individual patient data meta-analysis including almost 1,000 extremely preterm infants showed that early low-dose hydrocortisone therapy is beneficial for survival without BPD and without adverse effects on neurodevelopment at 2 years of age ^60^. Data from two clinical trials have shown that in very low-birth-weight infants with severe RDS, intra-tracheal administration of surfactant/budesonide combination compared with surfactant alone significantly decreased the incidence of BPD or death ^61, 62^. Larger trials are underway to determine if this should be recommended as standard of care. Mesenchymal stem cell (MSC) therapy and insulin-like growth factor (IGF)-1 (in combination with IGF-binding protein 3 [IGFB3]) are the new investigational agents that are being explored currently to prevent BPD. Figure 2 denotes the new and emerging therapies for the management and prevention of BPD.

### Stem cell-based therapies in bronchopulmonary dysplasia

MSCs play a vital role in lung development, and the therapeutic use of MSCs to treat and prevent BPD has emerged as a promising field. In preclinical studies, exogenous stem cells protect and repair hyperoxia-induced lung injury and improve survival. The precise mechanism by which they act is not completely known; however, it is believed that MSCs act primarily via a paracrine effect ^63^. In a systematic review evaluating the various cell‐based therapies in preclinical BPD, MSCs were found to be the most efficacious among all interventions, although they were exclusively investigated in the rodent hyperoxia-exposed model of BPD ^64^. Human umbilical cord tissue and cord blood have been identified as a potential source of MSCs and have been further explored as a feasible source of these cells and exosomes ^65, 66^. At least two phase I clinical trials of human cord blood-derived MSCs have been successfully conducted in South Korea and the United States ^67, 68^. Another source for similar cells is from placental membranes, human amnion epithelial cells (hAECs), which have shown considerable promise in preclinical models of BPD. The first human clinical trial of hAECs in neonates with BPD has been completed to assess the safety of these cells ^69^.

### Insulin-like growth factor-1

IGF-1 is a significant regulator of lung angiogenesis, fetal growth, and development. Lower levels of IGF-1 have been independently associated with increased risk of BPD and retinopathy of prematurity. In neonatal rodent models of hyperoxia-induced lung injury, the administration of IGF-1 seems to reduce lung injury ^70^. In a phase II clinical trial, treatment with recombinant human IGF along with its binding protein (rhIGF-1/rhIGFBP-3) reduced the incidence of severe BPD as a secondary outcome ^71^.

## Summary

Despite great advances in lung research and evolving management of BPD over the years, many questions remain unanswered and present unique opportunities for research. The pathobiology of BPD is a rapidly evolving field of research with potential for utilizing the newer “omic” approaches to better understand the molecular pathways and cell interactions specific to injury in BPD. The development of an appropriate BPD definition that is based on pathophysiology and can predict long-term pulmonary outcomes remains a top priority. This would also help as a useful and uniform clinical end point ^72^. As we continue on our search for better biomarkers in BPD, further research should focus on clinical, imaging, and biological markers to better diagnose and manage these infants. Lastly, the advancement in cell-based therapies provides a novel opportunity to understand the biology of the repair cells and identify new targets in injury and repair pathways in BPD. Despite the severe lung injury in BPD, most infants achieve remarkable recovery of lung structure and function. Exploring the cause of this resilience of the neonatal lung will provide further insights into the pathogenesis of chronic lung diseases.
